# 

**Published:** 1864-06

**Authors:** 


*Contents.*

ORIGINAL COMMUNICATIONS........................ 81

1. Prophylactic Effects of Quinine, by Surg. Samuel Logan. 2 Read's Case of Excision of Knee-Joint, reported by Assistant-Surgeon M. J. De Rosset. 3. Indigenous Medicinal Plants, by Assistant Surgeon P. C Grant. 4. Report of three cases illustrating the Correlation existing between Erysipelas, Diphtheria (and Hospital Gangrene?) by Assist. Surg. H. S. Chalmers. 5. A Case of Resection of Shoulder-Joint, by Surg. Wm. Alexander Greene. 6. The Effects of Minie Balls on Bone, by Surg. E. Lloyd Howard. Marriages of Consanguinity.

EDITORIAL AND MISCELLANEOUS........................ 89

Conservative Surgery in Compound Fracture of Femur.

TRANSACTIONS OF THE ASSOCIATION OF ARMY AND NAVY SURGEONS........................ 90

CHRONICLE OF MEDICAL SCIENCE........................ 95

1. Conservative Surgery in Compound Fracture of Femur (Gun-Shot Wounds), by M. Legouest. 2. On the Therapeutic Properties of Carbolic Acid, by Dr. Crace Calvert. 3. Records of Cases treated in St. Bartholomew's Hospital, by Surgeon Frederick C Skey. 4. The Natural Progress of Disease, by Dr. John Hugh Bennet.

